# Auxiliary Liver Graft Can Be Protected From HBV Infection in HBsAg Positive Blood Circulation

**DOI:** 10.3389/fmed.2021.726502

**Published:** 2021-08-25

**Authors:** Lin Wei, Hai-Ming Zhang, Chi-Dan Wan, Wei Qu, Zhi-Gui Zeng, Ying Liu, Jun Xiong, Li-Ying Sun, Zhi-Jun Zhu

**Affiliations:** ^1^Beijing Friendship Hospital, Capital Medical University, Beijing, China; ^2^Clinical Center for Pediatric Liver Transplantation, Capital Medical University, Beijing, China; ^3^National Clinical Research Center for Digestive Diseases, Beijing, China; ^4^Department of Hepatobiliary Surgery, Union Hospital, Tongji Medical College, Huazhong University of Science and Technology, Wuhan, China

**Keywords:** APOLT, entecavir, liver cirrhosis, liver regeneration, hepatitis B recurrence

## Abstract

Auxiliary grafts have a high risk of Hepatitis B virus (HBV) infection in patients with chronic HBV-related diseases. Hepatitis B virus-related auxiliary partial orthotopic liver transplantation (APOLT) cases were reviewed to show the results of current methods to block native-to-graft HBV transmission. Three patients received APOLT for HBV-related liver cirrhosis and a recurrent upper gastrointestinal hemorrhage between April 2015 and January 2017 by the liver transplant team of Beijing Friendship Hospital affiliated with Capital Medical University. All three patients were positive for HBV surface antigen (HBsAg) and had a negative HBV DNA test result before transplantation. After auxiliary transplantations, HBsAg was found to be positive in two patients and negative in one patient. To avoid graft infection of HBV, entecavir-based therapy was employed and the remnant native livers of the recipients were removed 51–878 days after liver transplantation. Then, serum conversions of HBsAg were found in all three cases. For the first time, this case series shows the possibility of blocking the transmission of HBV from a native liver to a graft in auxiliary transplantation by entecavir-based therapy. Among the cases, a left lobe graft was successfully implanted as a replacement of the right lobe of the recipient, which is also discussed.

## Introduction

In auxiliary partial orthotopic liver transplantation (APOLT), a liver graft is implanted while the partial or entire native liver is preserved. Two livers will co-function and support each other. Auxiliary partial orthotopic liver transplantation is commonly used in patients with acute liver failure or metabolic liver diseases (1, 2). Patients who receive APOLT for acute liver failure, a reversible liver disease, may be able to discontinue immunosuppressant treatment after their native liver has regenerated (3). Some inherent liver metabolic defects can be corrected by a small auxiliary graft as a special form of gene therapy (4, 5). Auxiliary partial orthotopic liver transplantation can also be employed when a whole liver graft cannot be acquired or the graft volume is inadequate (6, 7).

Hepatitis B virus (HBV)-related diseases are regarded as a contraindication for APOLT. The HBV-infected native liver is preserved in APOLT, which may potentially relieve HBV and increase the risk of graft infection. Hepatitis B virus recurrence after liver transplantation may progress rapidly and become difficult to control. Several reports showed attempts to carry out auxiliary liver transplantations in patients with HBV-related liver diseases. In the report of Onno, in 1988, four cases received a heterotopic liver transplantation for HBV-related liver cirrhosis, and immunofluorescence stains for the core antigen were positive in all of the grafts within 3 weeks after transplantation (8). One of these patients had clinical signs (recurrence of ascites). In 1991 and 1992, Kate FJ reported chronic hepatitis and cirrhosis in the auxiliary graft after auxiliary liver transplantation for HBV related liver diseases (9, 10). In the auxiliary liver transplantations for fulminant hepatitis B reported by Durand in 2002 (11), three patients who were positive for HBV surface antigen (HBsAg) received an auxiliary liver transplantation. Two patients survived, and one was followed up more than 1 year later. HBV surface antigen was found to be negative in these patients after the hepatitis B immune globulin (HBIG) treatment. However, in the only patient followed for more than 1 year, the HBIG titer was negative, which was most likely a residue of HBV. In 2008, Quaglia reported auxiliary liver transplantations for seronegative liver failure (12). Four cases of HBV infections were included. One of these patients died of HBV recurrence on day 1,505, and the other three patients lived (12). According to the results of these cases, the auxiliary graft conveyed a high risk of HBV infection in patients with chronic HBV-related diseases.

Great improvements in anti-HBV agents have been made. Nucleos(t)ide analogs and HBIG have been used as a combined therapy to prevent HBV recurrence after liver transplantation, which significantly reduces the HBV recurrence rate. In a report by Wang in 2017 (13), four patients with HBV-related liver cirrhosis received APOLT. Nucleos(t)ide analogs were administered after APOLT. After transplantation, all recipients were positive for serum HBsAg and negative for the HBsAg and HBV core antigen (HBcAg) in grafts. All patients lived and showed normal graft function within a mean follow-up period of 21 (13–26) months. However, immunostains for HBsAg and HBcAg at 1, 6, and 12 months after transplantation were not enough to exclude HBV infection in the auxiliary grafts. Whether the nucleos(t)ide analog blocked HBV infection should be further confirmed.

Because of insufficient donor graft volumes, three patients received APOLTs for HBV-related liver cirrhosis in our center. To avoid HBV infection of the auxiliary grafts, we removed the remnant native livers of the recipients 51–878 days after the liver transplantation. Then, serum HBsAg became negative in all three recipients. For the first time, this small case series shows that it is possible to completely block the transmission of HBV from the native liver to a graft in auxiliary transplantation by entecavir monotherapy or in combination with HBIG therapy.

## Patients and Methods

Three patients who received APOLTs for HBV-related liver cirrhosis were reviewed; These APOLTs were conducted by the liver transplant team of Beijing Friendship Hospital affiliated with Capital Medical University. This study and the APOLT treatment were approved by the ethics committee of Beijing Friendship Hospital. Informed consent for the operations and this study was obtained. All three patients received APOLT for HBV-related liver cirrhosis and recurrent upper gastrointestinal hemorrhage. Living donor liver transplantation was the only option for these patients due to the shortage of organs (14). However, the left lobe of each donor liver was too small to be transplanted, with a graft-to-recipient weight ratio (GRWR) less than 0.8%. Additionally, the right lobe could not be donated because the volume of the remnant liver was insufficient to ensure the donor's safety because the proportion was <35% (Table 1). Thus APOLT was employed to prevent small for size syndrome (SFSS).

**Table 1:** Volumetric assessment of left/right lobe of living donor by computerized tomography.

**Donor and patient**	**Potential graft**	**Volume of graft (ml)**	**RLV/WLV (%)**	**Body weight of recipient (kg)**	**GRWR (%)**	**Conclusion**
Donor of patient A	Left lobe	396	69.9	68	0.58	Insufficient graft
	Right lobe	921	30.1		1.35	Insufficient remnant liver
Donor of Patient B	Left lobe	448	68.0	76	0.59	Insufficient graft
	Right lobe	952	32.0	76	1.25	Insufficient remnant liver
Donor of Patient C	Left lobe	390	72.7	73	0.53	Insufficient graft
	Right lobe	1,041	27.3	73	1.42	Insufficient remnant liver

*RLV, remnant liver volume; WLV, whole liver volume; GRWR, graft recipient's body weight ratio*.

**Figure 1:**
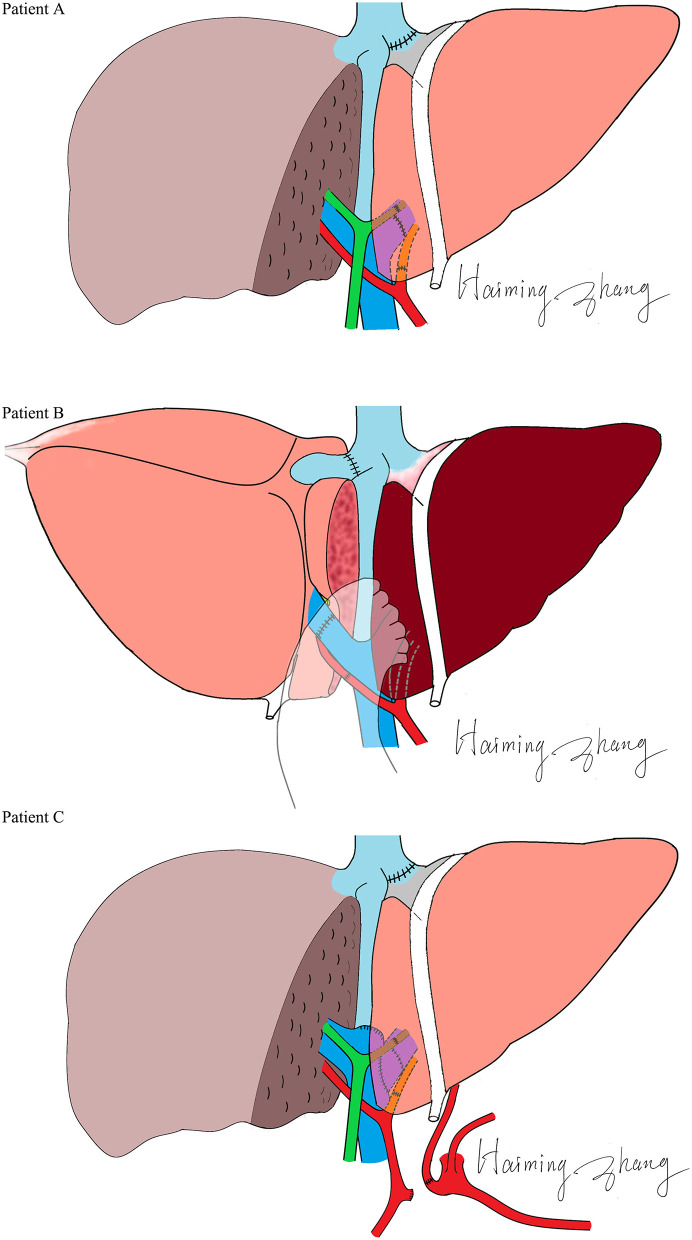
Methods of auxiliary graft reconstructions.

The characteristics of the donors and recipients are provided in Table 2. Patient A was 55 years old and was admitted for a recurrent upper gastrointestinal hemorrhage. His first upper gastrointestinal hemorrhage was found on January 6^th^, 2015, and then, splenectomy and disconnection were performed in 2007. Patient C, a 52-year-old man, was admitted with the same diagnosis and no surgical history. Both of these patients received left lobe APOLT by replacing the left lobes of their native livers on April 26^th^, 2015, and January 11^th^, 2017, respectively. Patient B was 29 years old. The patient experienced an upper gastrointestinal hemorrhage in 2010. Splenectomy and disconnection were conducted to prevent recurrent hemorrhage. In November 2011, the patient suffered from a severe upper gastrointestinal hemorrhage again. Although endoscopic sclerotherapy was performed, it did not stop recurrent hemorrhage. APOLT was planned. Abdominal adhesions due to previous surgery limited the space of the upper left abdominal cavity. Finally, we implanted the left lobe graft into the upper right abdominal cavity of the recipient after removing the right lobe of his native liver on August 16^th^, 2016 (Figure 1).

**Table 2:** Characteristics of donors and recipients in auxiliary liver transplantation.

	**Patient A**	**Patient B**	**Patient C**
**Recipient**			
Age (year)/sex	55/male	29/male	52/male
Cause of liver cirrhosis	Hepatitis B	Hepatitis B	Hepatitis B
ABO blood group	A	B	O
Serum result of HBV	HBsAg(+) HBsAb(–) HBeAg(–) HBeAb(–) HBcAb(+)	HBsAg(+) HBsAb(–) HBeAg(–) HBeAb(+) HBcAb(+)	HBsAg(+) HBsAb(–) HBeAg(–) HBeAb(+) HBcAb(+)
Child grade	6	6	7
MELD score	11	9	11
Platelet count	102 × 10^9^/L	382 × 10^9^/L	45 × 10^9^/L
Albumin (g/L)	33.3	38.0	43.1
Total bilirubin (μmol/L)	22.0	18.87	37.96
INR	1.43	1.18	1.18
Creatinine (lmol/L)	66.0	67.5	73.1
Serum sodium (lmol/L)	138.9	139	140.8
Ascites	No	No	mild
Esophageal and gastric varices	Severe	Severe	Severe
**Donor**			
Age (year)/sex	45/female	50/male	47/female
Serum result of HBV	HBsAg(–) HBsAb(+) HBeAg(–) HBeAb(–) HBcAb(+)	HBsAg(–) HBsAb(+) HBeAg(–) HBeAb(–) HBcAb(–)	HBsAg(–) HBsAb(+) HBeAg(–) HBeAb(–) HBcAb(–)
**Graft**			
Type of graft (lobe)	Left lobe (without middle hepatic vein)	Left lobe (without middle hepatic vein)	Left lobe (without middle hepatic vein)
Graft weight during operation (g)	386	408	349
ABO blood group	A	O	O
Cold ischemic time	2 h 5 min	6 h 15 min	4 h 10 min
Warm ischemic time	1 min	1 min	1 min
GW/RW	0.57%	0.54%	0.46%
Duration of operation	9 h 36 min	13 h 50 min	12 h 20 min
Blood loss (ml)	200	3,000	2,400
Post-operative complications	Anatomotic stricture	None	Anatomotic stricture
Follow-up (days)	1,200	566	525

Left lobes without the middle hepatic vein were procured as grafts in all of these APOLTs. In patient A, the left hepatic vein, left portal vein and left hepatic artery were anastomosed to their corresponding parts of the recipient. In patient B, the left hepatic vein, left portal vein, and left hepatic artery were anastomosed to the right hepatic vein, right portal vein, and right hepatic artery of the recipient. The left hepatic duct was reconstructed by biliary-enterostomy in patient B. In patient C, the portal vein (PV) of the graft was elongated by a vein conduit taken from the left portal vein of the recipient and anatomized to the recipient's PV by the end to side method. The left hepatic artery of the graft was reconstructed to the left hepatic artery of the recipient. An auxiliary left hepatic artery was also found in patient C. The common hepatic artery of patient C was transected. The proximal end was connected to the auxiliary left hepatic artery of the graft, and the distal end was closed. End to end biliary anastomoses were performed in patients A and C.

After APOLT, the biochemistry results showed normal liver function in all three patients. The volumes of the grafts increased over time, and atrophy was found in the remnant native livers. We removed the remnant native livers at 51–878 days after transplantation to prevent HBV infection (patients A, B, and C at 878, 136, and 51 days, respectively).

Antibiotics, immunosuppressant, and antithrombotic agents were administered. Tacrolimus, mycophenolate mofetil, and steroids were included in the immunosuppression regimen. The target serum level of tacrolimus was 6 and 8 ng/ml. Entecavir was administered continuously since APOLT. Hepatitis B immune globulin was not given to patient A. Patient B and patient C received 4,000 IU HBIG during APOLT and 2,000–4000 IU HBIG once per day within the first week after transplantation. If the patient was positive for the HBV surface antibody (HBsAb), HBIG was given according to the blood titer. Hepatitis B immune globulin therapy was not continued if a patient was persistently positive for HBsAg and negative for HBsAb. In patient B and patient C, 4,000 IU HBIG was also given during the hepatectomy, and then, HBIG was administered based on the blood titer.

Routine blood tests, biochemical markers for liver and kidney functions, and ultrasonography were conducted at the early stages, after transplantation and at each follow-up. Computed tomography (CT) was used to estimate the volumes of the grafts and native livers, if necessary. Liver biopsies were performed during the hepatectomies.

## Results

### Graft Function and Complications

**Figure 2:**
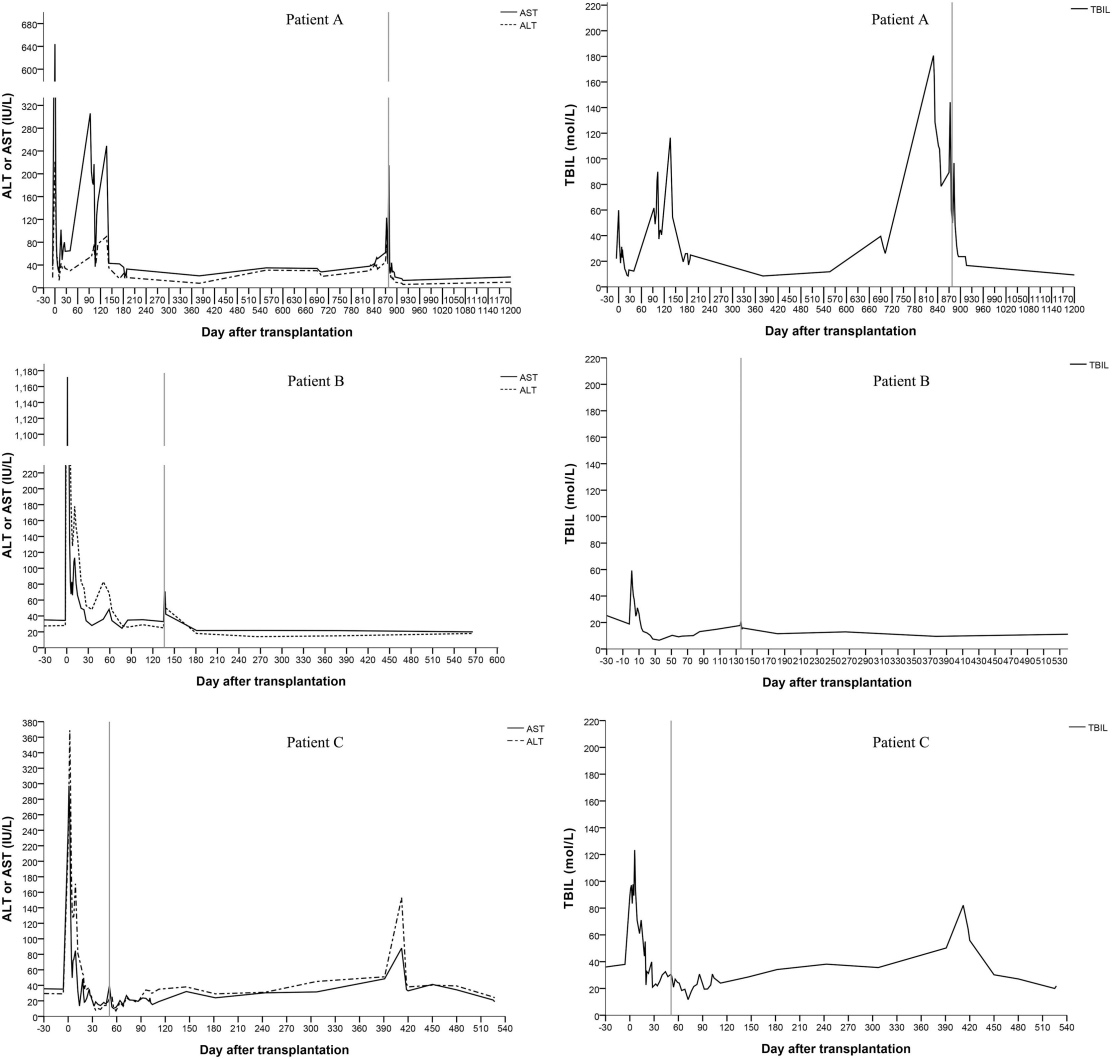
Changes of the serum biochemical parameters for liver function (the vertical line shows the date of the hepatectomy of the remnant native liver).

All three patients recovered from the operations, and their liver functions were normal at the end of the follow-up. The duration of the intensive care unit (ICU) stay was 3, 2 and 2 days for patients A, B, and C, respectively. No serious infections or surgical complications were found. Antibiotics were stopped 5–7 days after transplantation. Patients were discharged 21–28 days after transplantation. Patient A showed elevated levels of alanine aminotransferase (ALT), aspartate aminotransferase (AST), and total bilirubin (TBIL) 136 days after transplantation. Magnetic resonance cholangiopancreatography (MRCP) showed a biliary anastomotic stricture, and a percutaneous transhepatic cholangiodrainage (PTCD) was placed. Then, the biochemical parameters returned to normal. However, choledocholithiasis occurred 829 days after transplantation. Choledoenterostomy was performed during the hepatectomy at the 878^th^ day after transplantation. Finally, the biochemical parameters of the liver function became normal. Patient B recovered smoothly; no complications were found during follow-up. A biliary anastomotic stricture was also found in patient C 391 days after transplantation, which was treated successfully by PTCD. No serious infections, acute rejections or chronic rejections were found among these patients. According to the results of ultrasonography, the artery, PV, and hepatic vein of the grafts were patent and the blood flow velocities were within the normal range. The levels of ALT, AST, and TBIL are shown in Figure 2.

### HBV Markers

**Figure 3:**
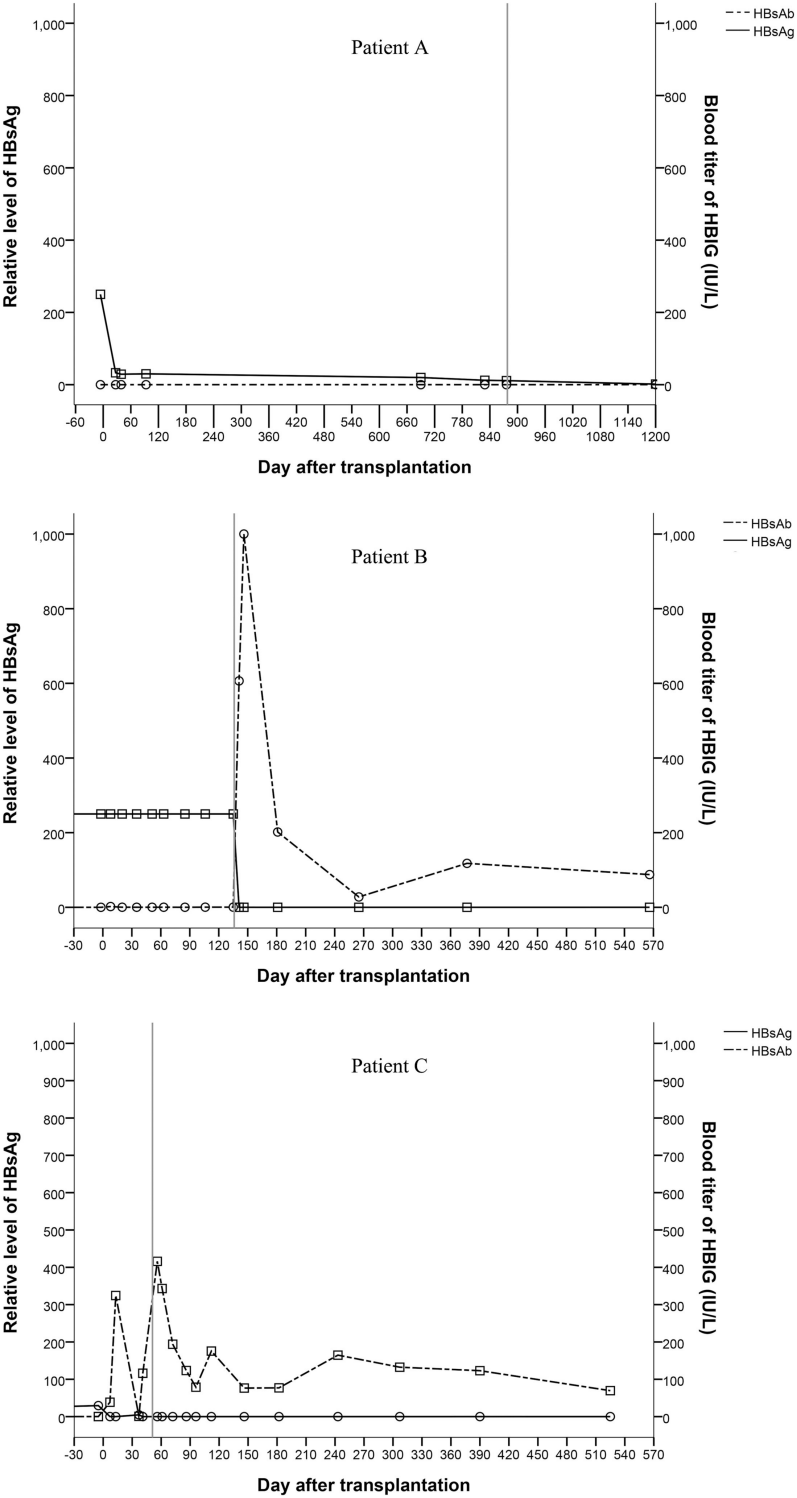
Changes of serum markers for HBV (the vertical line shows the date of the hepatectomy of the remnant native liver).

All recipients were positive for serum HBsAg before liver transplantation. Patient A and patient B were HBsAg positive after transplantation even after receiving entecavir and HBIG therapy. Patient C showed unstable HBsAg and HBsAb levels after transplantation. All three patients were HBsAg negative and HBsAb and HBV core antibody (HBcAb) positive after hepatectomy (Figure 3). Three hundred and twenty-two days after the complete removal of his native liver, patient A was negative for all of the serum markers of HBV, including HBsAg, HBsAb, HBV e antigen (HBeAg), HBV e antibody (HBeAb), HBcAb, and pre S1 antigen (Pre-S1Ag). Patient B and patient C were followed for 566 and 525 days, respectively, and no changes in the serum markers of HBV were found since hepatectomies. All three patients were negative for HBV DNA before and after transplantation, which was tested every 2 weeks after transplantation until discharge after hepatectomy. Biopsies were taken from the grafts during hepatectomy. Immunostaining of donor liver tissues for HBsAg and HBcAg were all negative.

### Graft Volume

**Figure 4:**
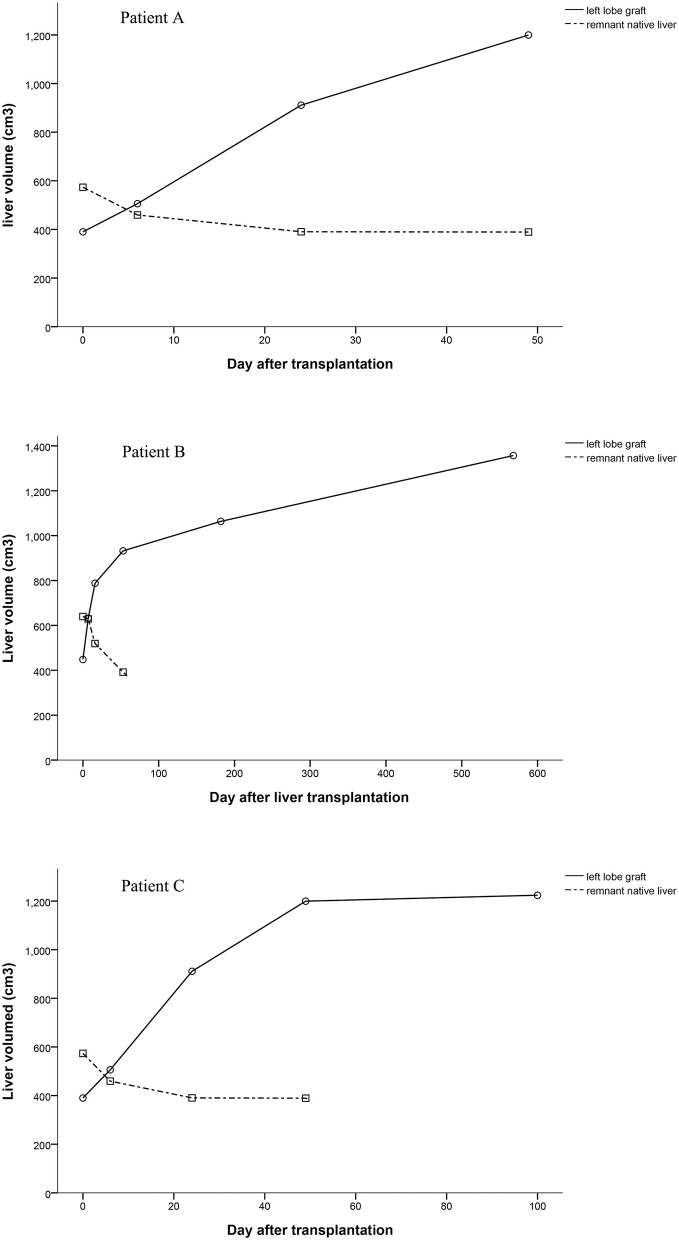
Volume changes of the auxiliary grafts and native liver.

Computed tomography data of patients could be found at several time points. We calculated volumes of grafts and native livers by the available Computed tomography data and showed them in Figure 4. The volumes of the grafts increased over time, especially in the early stages after liver transplantation (Figure 4). Since the remnant native livers were cirrhotic, the blood supply to them was very low, which was detected by ultrasonography but could not be quantified. The volume of the remnant native liver continued to reduce as a result of portal blood competition with the graft (Figure 4). The trend of the PV blood velocity changes was roughly matched with the total volume changes of grafts. However, the PV blood velocities varied greatly at the early stages after transplantation, and the low density of examinations at the late stages cannot show a clear trend of PV blood velocity changes.

## Discussion

In these three APOLT recipients, the remnant native livers releasing HBsAg may be potential sources of HBV. However, the liver grafts were not infected, which can be supported by HBsAg conversions after removing native livers. Thus, in these APOLTs, entecavir completely blocked HBV transmission from the HBV-infected native livers to the grafts, even though the two partial livers shared blood circulation for 51 to 878 days. The combination of lamivudine plus HBIG is the most widely used treatment to prevent HBV recurrence after liver transplantation for HBV-related diseases. Lamivudine alone has been shown to be insufficient to completely block the transmission of HBV to the HBV-free graft after liver transplantation (15). New nucleos(t)ide analogs have been used and showed high efficacy in preventing HBV recurrence (16). Entecavir is also used as monotherapy to prevent HBV recurrence (17). However, it is not clear whether HBV transmission can be completely blocked by nucleo(t)ide analogs in HBV-related APOLT. In classic liver transplantation, the HBV infected liver is completely removed. Thus, entecavir can block HBV infection after the serum conversion of HBsAg. In auxiliary liver transplantation, part of the HBV-infected liver continues to release HBsAg. The auxiliary graft seems to inevitably become infected by HBV if entecavir cannot completely prevent viron formation. Liver transplant recipients are immuno-compromised, which also increases the risk of HBV infection. Previous clinical studies (see the introduction) of HBV-related auxiliary liver transplantation showed that auxiliary grafts were normal in function and negative in immunological stains for HBsAg and HBcAg after entecavir administration, which suggested the practical of APOLT for HBV related diseases. Our cases further demonstrated the possibility of preventing graft infection of HBV in auxiliary liver transplantation, especially in patient A whose native liver was removed 878 days after transplantation. By removing the remnant native liver, HBV infection can be cured after APLOT. Keeping grafts free from HBV infection should be emphasized in the process of auxiliary liver transplantation. In the current series, only patients with liver cirrhosis received APOLT. The blood flow resistance in the cirrhotic native liver increased dramatically compared to that in the grafts. Thus, portal blood flow of the native livers was too low to be quantified, and most of the portal blood supply went to the grafts. Fast increases in the graft volumes were found, together with atrophy of the native livers (Figure 4). In functional respects, the remnant native livers contributed little to the total liver function and could be safety removed at relative early stages. Therefore, we removed the remnant native liver of patient C only 51 days after transplantation to reduce the graft exposure to HBsAg positive circulation. Thus, effective anti-HBV treatment and early removal of the remnant liver are suggested in APOLT for HBV-related liver cirrhosis to reduce the risk of HBV infecting grafts.

A special manner of left lobe graft implantation was used in patient B. The left lobe graft was implanted instead of the right lobe of the recipient. This successful case suggested the practicability of this operation manner, which may be a promising way to settle the space issue in left lobe implantation. Abdominal adhesions resulting from previous surgeries are a common problem, as splenectomies with disconnection are conventional treatments. By reconstructing the normal anatomical structure, a more stable blood flow can be maintained. As reported, orthotopic auxiliary liver transplantation may lead to fewer complications than heterotopic transplantation (18). Therefore, transplant surgeons usually make more effort to dissect adhesions rather than change the position of a graft. However, when we rolled the left lobe over, it was well matched with the right lobe in regard to the portal and hepatic veins. The risks of angulation, compression and twist can also be reduced by precise vessel reconstruction. No vessel graft was used in this case. In an APOLT for non-cirrhotic liver metabolic disease, a reduced left lobe graft blood supply was found (18). The relatively high blood flow resistance due to small volume, immunological, and ischemic injuries may result in a continuing reduction of the blood supply of the left graft, especially when in competition with the non-cirrhotic native liver. Placing the left lobe in the right side may contribute to the balance of the PV blood distribution because of the similar volumes of the donor and remnant native livers. More blood supply to a donor from the right branch of the PV may balance the risk of immune and ischemia injuries. Thus, we believe that this technique may have a special advantage in APOLTs for non-cirrhotic liver diseases.

No complications of the blood vessels were found in any of the recipients. Anastomoses of the PVs were carefully designed in these cases. In patient C, the left PV of the graft was reconstructed by the end to side method and the angulation of end to end anastomosis was avoided. The anastomosis of the PV was pushed to the opposite direction of the graft with the increasing volume of the graft after transplantation. This change should be taken into consideration before PV reconstruction. When the end of the donor's (left or right) PV is lower than the bifurcation of the recipient's PV, the end to side method was suggested. Biliary anastomotic strictures were found in the two cases that received end to end biliary anastomosis. Though an improvement was found after PTCD, choledochojejunostomy seemed to be an optimal way to prevent or treat this problem. All patients recovered and had a normal liver function.

As reported (6, 7, 19) previously, current practice also supports auxiliary liver transplantation as an aid for a SFSS graft. However, in patients with severe liver cirrhosis, the function and PV blood flow of the liver decline to a large degree and a relatively large graft would be needed even for auxiliary liver transplantation. In these patients, hepatic failure, PV hypertension, and blood coagulation disorders may also increase the risk and complications of APOLT. Therefore, only patients with relatively mild cirrhosis and an indication of a gastrointestinal hemorrhage were considered for APOLT in the current series. Right lobe and whole liver grafts would be preferred for patents with more severe cirrhosis.

## Conclusion

It is possible to block the transmission of HBV between the native liver and graft by a sensitive nucleos(t)ide drug treatment. Auxiliary transplantation of left lobe grafts may be a practical option for recipients at risk for SFSS. Left lobe grafts can also be implanted into the space of the right lobe of the liver safely.

## Data Availability Statement

The original contributions presented in the study are included in the article/supplementary material, further inquiries can be directed to the corresponding author/s.

## Ethics Statement

The studies involving human participants were reviewed and approved by the ethics committee of Beijing Friendship Hospital. The patients/participants provided their written informed consent to participate in this study.

## Author Contributions

L-YS: participated in the research design and the patient management. Z-JZ: planned and performed the operations and participated in operations. JX and YL: participated in the patient management after the operations. WQ: participated in operations. C-DW: contributed to treatments and operations as an expert consultant. H-MZ: participated in data analysis and the writing of the paper. LW: participated in the research design and the surgical process of operations. All authors contributed to the article and approved the submitted version.

## Conflict of Interest

The authors declare that the research was conducted in the absence of any commercial or financial relationships that could be construed as a potential conflict of interest.

## Publisher's Note

All claims expressed in this article are solely those of the authors and do not necessarily represent those of their affiliated organizations, or those of the publisher, the editors and the reviewers. Any product that may be evaluated in this article, or claim that may be made by its manufacturer, is not guaranteed or endorsed by the publisher.

## Abbreviations

APOLT: auxiliary partial orthotopic liver transplantation
HBV: Hepatitis B virus
HBsAg: HBV surface antigen
HBIG: Hepatitis B immune globulin
HBcAg: HBV core antigen
GRWR: graft-to-recipient weight ratio
SFSS: small for size syndrome
HBsAb: HBV surface antibody
ICU: intensive care unit
ALT: alanine aminotransferase
AST: aspartate aminotransferase
TBIL: total bilirubin
MRCP: magnetic resonance cholangiopancreatography
PTCD: percutaneous transhepatic cholangiodrainage
HBeAg: HBV e antigen
HBeAb: HBV e antibody
Pre-S1Ag: pre S1 antigen
PV: portal vein.
